# Gene Transfer of Mutant Mouse Cholinesterase Provides High Lifetime Expression and Reduced Cocaine Responses with No Evident Toxicity

**DOI:** 10.1371/journal.pone.0067446

**Published:** 2013-06-28

**Authors:** Liyi Geng, Yang Gao, Xiabin Chen, Shurong Hou, Chang-Guo Zhan, Zoran Radic, Robin J. Parks, Stephen J. Russell, Linh Pham, Stephen Brimijoin

**Affiliations:** 1 Department of Molecular Pharmacology and Experimental Therapeutics, Mayo Clinic, Rochester, Minnesota, United States of America; 2 Department of Pharmaceutical Sciences, College of Pharmacy, University of Kentucky, Lexington, Kentucky, United States of America; 3 Skaggs School of Pharmacy and Pharmaceutical Sciences, University of California San Diego, LaJolla, California, United States of America; 4 Regenerative Medicine Program, Ottawa Hospital Research Institute, Ottawa, Ontario, Canada; 5 Department of Molecular Medicine, Mayo Clinic, Rochester, Minnesota, United States of America; Max Planck Institute of Psychiatry, Germany

## Abstract

Gene transfer of a human cocaine hydrolase (hCocH) derived from butyrylcholinesterase (BChE) by 5 mutations (A199S/F227A/S287G/A328W/Y332G) has shown promise in animal studies for treatment of cocaine addiction. To predict the physiological fate and immunogenicity of this enzyme in humans, a comparable enzyme was created and tested in a conspecific host. Thus, similar mutations (A199S/**S227**A/S287G/A328W/Y332G) were introduced into mouse BChE to obtain a mouse CocH (mCocH). The cDNA was incorporated into viral vectors based on: a) serotype-5 helper-dependent adenovirus (hdAD) with ApoE promoter, and b) serotype-8 adeno-associated virus with CMV promoter (AAV-CMV) or multiple promoter and enhancer elements (AAV-VIP). Experiments on substrate kinetics of purified mCocH expressed in HEK293T cells showed 30-fold higher activity (U/mg) with ^3^H-cocaine and 25% lower activity with butyrylthiocholine, compared with wild type BChE. In mice given modest doses of AAV-CMV-mCocH vector (0.7 or 3×10^11^ particles) plasma hydrolase activity rose 10-fold above control for over one year with no observed immune response. Under the same conditions, transduction of the human counterpart continued less than 2 months and antibodies to hCocH were readily detected. The advanced AAV-VIP-mCocH vector generated a dose-dependent rise in plasma cocaine hydrolase activity from 20-fold (10^10^ particles) to 20,000 fold (10^13^ particles), while the hdAD vector (1.7×10^12^ particles) yielded a 300,000-fold increase. Neither vector caused adverse reactions such as motor weakness, elevated liver enzymes, or disturbance in spontaneous activity. Furthermore, treatment with high dose hdAD-ApoE-mCocH vector (1.7×10^12^ particles) prevented locomotor abnormalities, other behavioral signs, and release of hepatic alanine amino transferase after a cocaine dose fatal to most control mice (120 mg/kg). This outcome suggests that viral gene transfer can yield clinically effective cocaine hydrolase expression for lengthy periods without immune reactions or cholinergic dysfunction, while blocking toxicity from drug overdose.

## Introduction

Recent work in several laboratories has addressed the possibility of treating cocaine addiction with “interceptor proteins” that block cocaine’s access to brain reward centers [1]. The aim of such a therapy would be to reduce the risk of relapse into drug-taking provoked by cocaine re-encounter. This could be accomplished by antibody binding (via vaccination) or by metabolic destruction (via enzyme delivery). We have focused on the latter approach, specifically using viral gene transfer to deliver a human butyrylcholinesterase (BChE) optimized for cocaine hydrolysis by previously reported active-site mutations [2], [3], [4]. The general feasibility of using this cocaine hydrolase (CocH) was established in rat studies showing that CocH gene transfer can suppress drug-primed reinstatement of cocaine-seeking behavior for at least six months [5]. Further work indicates that a combined therapy using anti-cocaine vaccine along with CocH gene transfer might be even more effective than either single treatment [6].

As these studies were progressing, research focused on natural human BChE as a prophylactic against chemical warfare agents showed that this enzyme is physiologically benign [7], [8], [9]. In accord, we have not yet seen any toxicity in mice or rats receiving CocH injections or expressing CocH after viral gene transfer. Our accumulated data do indicate, however, that rodents develop antibodies against human BChE and CocH, which speed clearance of these transduced proteins and lower their plasma levels. This is to be expected because human BChE shares only 80% sequence identity with its rodent counterparts [10]. Antibody responses should be less likely in patients undergoing an enzyme-based therapy for cocaine abuse because only five amino acid residues distinguish CocH from natural BChE. Nonetheless, if such reactions did occur, they would curtail or impair the effect of treatment. Our chief goal in the present study, aiming toward a future clinical trial of gene transfer, was to determine whether the particular mutations conferring high activity against cocaine as a substrate could constitute an immunologic stimulus. Such an outcome would justify increased caution regarding safety and efficacy. A second goal, also in preparing for application in humans, was to begin comparing two different vector platforms in terms of efficacy and potential toxicity. Finally, to address a speculative concern, a last goal was to determine whether high plasma levels of CocH might impair cholinergic function by virtue of the enzyme's retained ability to hydrolyze acetylcholine,.

In order to evaluate these issues in an animal model, we generated a comparable cocaine hydrolase based upon *murine* BChE (mCocH) and incorporated it into viral gene transfer vectors for testing in mice. Here we report: 1) catalytic properties of mCocH with cocaine and the “conventional” BChE substrates, acetylcholine and butyrylthiocholine; 2) enzyme plasma levels and turnover rate after transduction *in vivo* by adeno-associated and helper-dependent adenoviral vectors using different promoter systems; 3) duration of expression and tests of antibody responses; 4) effect on locomotor responses to cocaine administration; and 5) liability for toxicity or tissue damage as reflected in levels of sentinel enzymes from liver, heart, and skeletal muscle.

## Results

### Enzyme Substrate Kinetics

**Figure 1:**
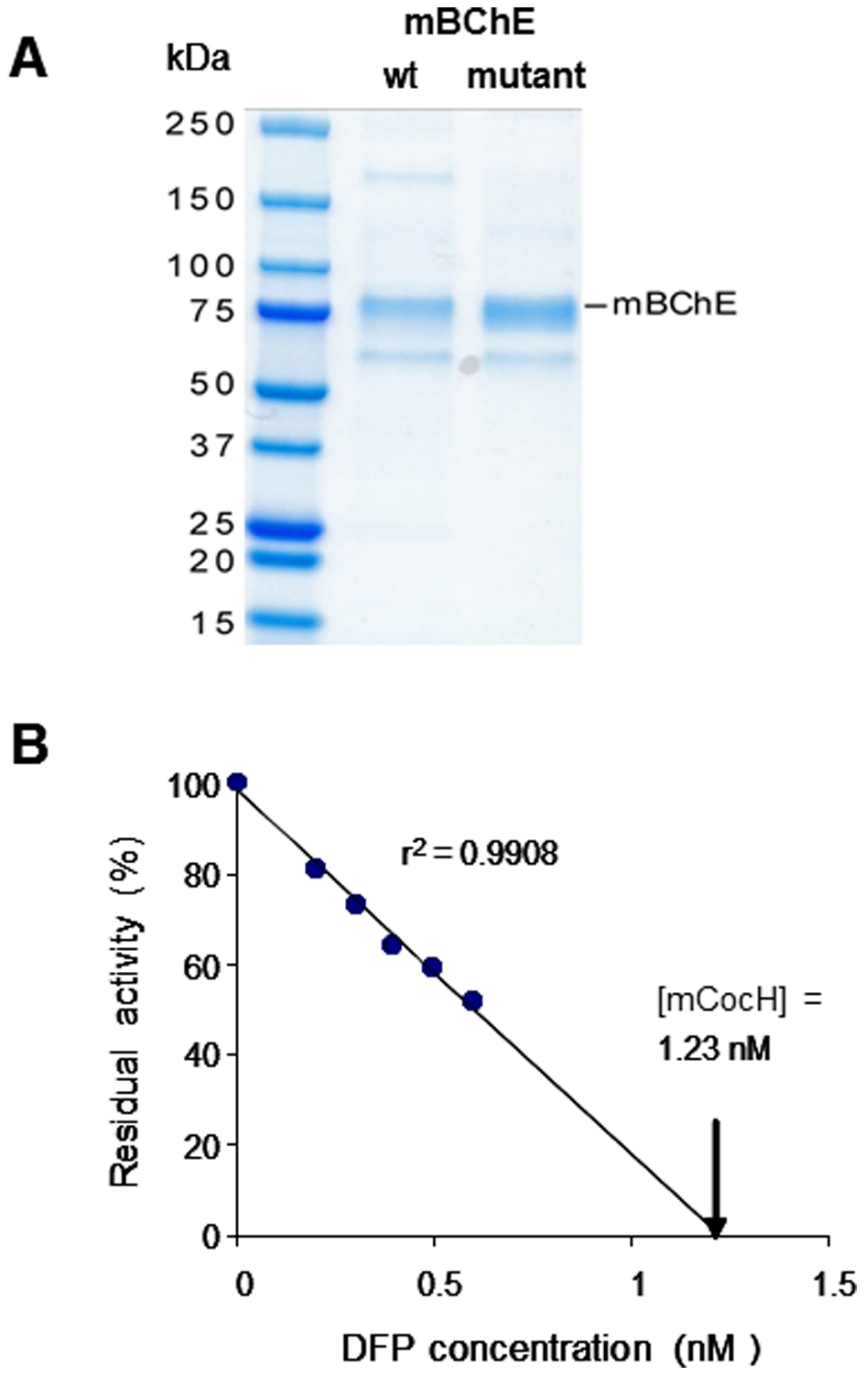
Purification of murine BChE (mBChE). A) SDS-PAGE gel with single major protein bands after purification of wild type mouse enzyme and the cocaine hydrolase mutant (mCocH); B) Active-site titration of mCocH with diisopropylfluorophosphate.

Mutated mouse BChE cDNA, A199S/S227A/S287G/A328WY332G (mCocH), was incorporated into adeno-associated viral vector (AAV8) and helper-dependent adenoviral vector (hdAD) as described in METHODS. To obtain protein for enzyme kinetics studies, HEK293T cells were transfected with AAV shuttle plasmid and enzyme was purified from culture supernatants. After active site titration with DFP (Fig. 1), a study of enzyme kinetics with ^3^H-cocaine, butyrylthiocholine, and acetylcholine as substrates was carried out. The results (Table 1) showed that mCocH had nearly 10-fold greater catalytic efficiency with cocaine (k_cat_/Km = 7.1) than wild-type mouse BChE (k_cat_/Km = 0.86). In contrast, mCocH showed reduced efficiency with butyrylthiocholine: apparent Km and Vmax = 99 µM and 330 U/mg, compared with 72 µM and 420 U/mg for wild type enzyme. The mutated enzyme also exhibited definite substrate inhibition instead of the substrate activation usual with wild-type BChE. Thus the altered amino acid residues appear to have created a secondary binding site for butyrylthiocholine that did not lead to hydrolysis but blocked access to the active site. Possible structural explanations for this effect are under investigation.

**Table 1:** Summary of kinetic constants for hydrolysis of cocaine, acetylcholine (ACh), and butyrylthiocholine (BThCh) by wild type mouse BChE as previously determined [14], [15] and mCocH, determined as described in Methods.

	WT mBChE			mCocH		
	BThCh	ACh	Cocaine	BThCh	ACh	Cocaine
**Km** (µM)	72	400	1.6	99	210	35
**Vmax** (U/mg)	420	460	0.01	330	230	3.0
**k_cat_** (min^−1^)	35,600	38,400	1.4	28,200	19,000	250
**k_cat_/Km**	490	96	0.86	285	90	7.1

Given constants are mean values of three or more experiments. Standard error of determination was typically 30% or less of the constant value.

### Indefinitely Sustained Transduction After Low dose Viral Vector

**Figure 2:**
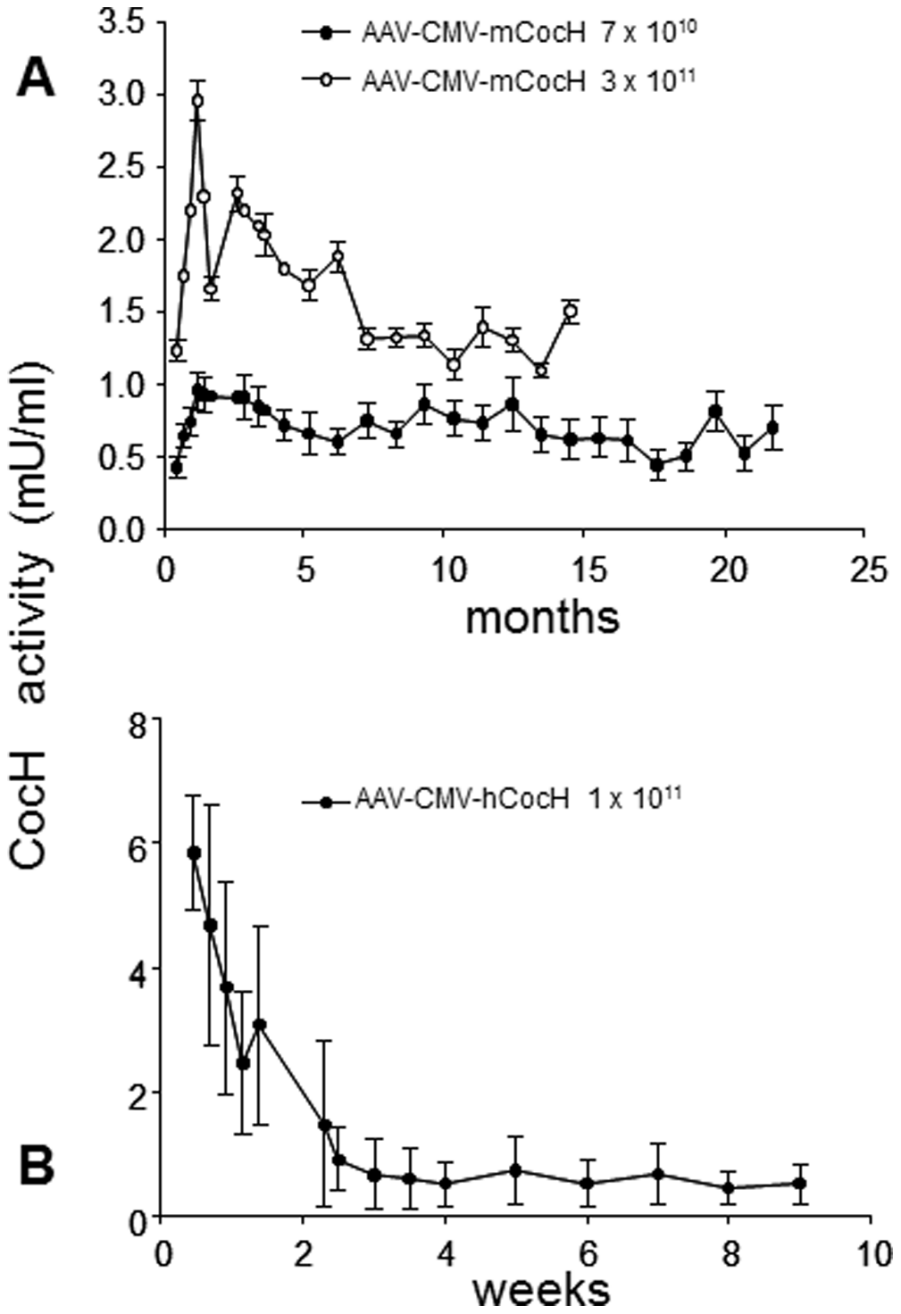
Expression of mouse and human CocH with AAV vector. A). Mice were treated with either of two different i.v. doses of AAV-CMV-mCocH vector: 7×10^10^ viral particles (filled circles, n = 4) or 3×10^11^ viral particles (open circles, n = 12). Other mice were given empty vector or saline treatment. Shown are cocaine hydrolase activities (mU/ml) in plasma samples at the indicated times (data from control mice are not represented because cocaine hydrolysis was barely measurable, less than 0.1 mU/ml). Note duration approaching 2 years. B). AAV-CMV-hCocH vector (10^11^ viral particles) was administered (n = 10), and cocaine hydrolase activities were monitored during the comparatively brief expression (time scale in *weeks*).

Although mCocH was 100-fold less efficient than human CocH at hydrolyzing cocaine [2], [3], a gene transfer still raised plasma cocaine hydrolase activity at least an order of magnitude. This effect enabled us to examine the duration of transduction as well as the protein turnover rate and plasma half-life, key measures of *in vivo* stability. For the purpose, groups of four and twelve mice, respectively, were given AAV-CMV-mCocH vector in i.v. doses of 0.7 or 3×10^11^ viral particles, and blood samples were tested periodically for cocaine hydrolase activity. The activity from endogenous mouse BChE (pre-injection or untreated control samples) was 0.1 mU/ml, but in vector-treated animals, cocaine hydrolysis rose 10 to 30 fold over the next two weeks (Fig. 2A). Furthermore, elevated levels continued indefinitely with only slight reduction over an extended period (respectively 15 and 22 months to date, still continuing). Thus, in practical terms, gene transfer supported near-lifetime expression of the mutant hydrolase. In contrast, 10 mice transduced with *human* CocH by the same AAV-CMV vector platform (10^11^ viral particles) expressed that foreign enzyme for only a few weeks (Fig. 2B). This curtailed expression was associated with immunological responses (see below), which may have accelerated the enzyme’s degradation.

### Molecular Forms and Plasma Half-life of Transduced mCocH

**Figure 3:**
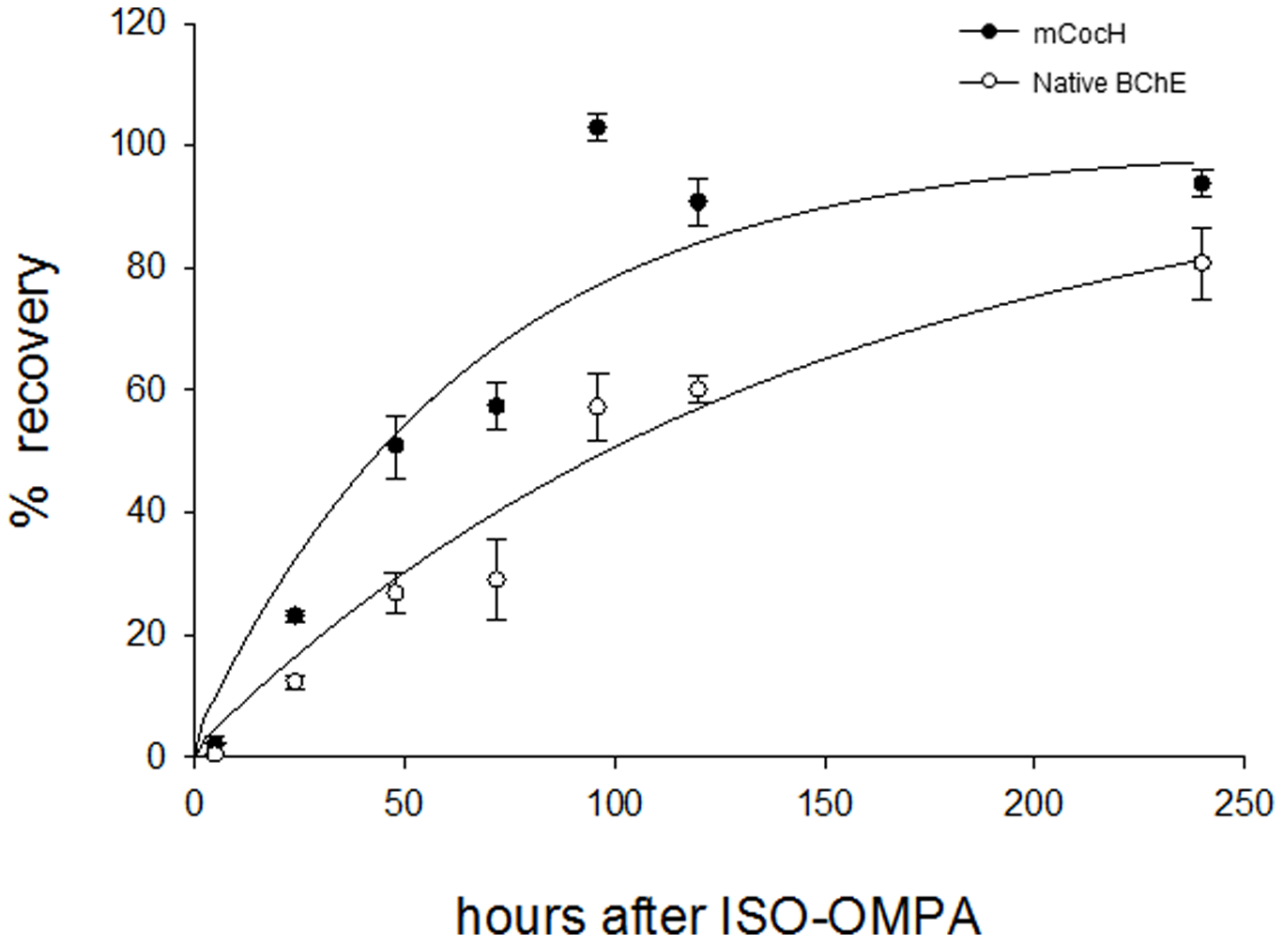
Turnover of vector-delivered mCocH and native BChE. Mice treated 5 months beforehand with 3×10^11^ particles of AAV vector encoding mCocH, and mice with no prior treatment, received 50 mg/kg of iso-OMPA at time zero, causing ∼95% inhibition of plasma CocH/BChE activities. Time-course of recovery was monitored as an index of replacement by newly synthesized enzyme. Relative rates are shown in activities as percent of pre-treatment levels.

On ultracentrifugation native mouse BChE sediments mainly as an 11S tetramer generated by interaction between proline-rich attachment domains (PRAD) and small polyproline peptides [11]. However, in a separate study (Geng, Gao, Brimijoin and Zhan, unpublished data) transduced mouse CocH sedimented as a 6.5 S dimer. Since BChE dimers are typically less stable than BChE tetramers, we attempted to estimate the *in vivo* turnover rate of the transduced protein from the recovery of enzyme activity after irreversible inhibition. This inhibition was accomplished with the organophosphate anticholinesterase, iso-OMPA, highly selective for BChE’s active site serine residue. Mice were given iso-OMPA (50 mg/kg, i.p.) approximately 6 weeks after injection of AAV-CMV-mCocH vector (3×10^11^ particles) or saline solution. In both groups, iso-OMPA decreased BChE activity by 95% within 5 hr, without indications of cholinergic toxicity. Because *in vitro* tests indicated that enzyme in plasma samples directly exposed to iso-OMPA did not reactivate spontaneously during prolonged dialysis (up to 48 hr), we used the time course of *in vivo* recovery as an index of replacement by newly synthesized enzyme (Fig. 3). The t_1/2_ for recovery of mouse CocH activity in vector-treated mice was 46±10 hr. In comparison native BChE activity in control mice recovered with t_1/2_ = 110±25 hr. Thus, the mutated enzyme was slightly less stable *in vivo* than the native protein. The greatly elevated enzyme level and slightly reduced enzyme half-life imply a very large increase of BChE synthesis in vector-treated mice (in mg of enzyme protein/day).

### Low Immunogenicity of mCocH

One factor that might shorten the *in vivo* half-life of mCocH would be an immune response. To address that possibility, blood sampled from vector-transduced mice across a range of times was screened for antibodies against mCocH. The tests (see Methods) utilized an assay previously found to have high specificity and sensitivity to detect enzyme-directed immunoglobulins, both IgG and IgM [12]. Positive control samples were taken from mice transduced with human CocH, which was expected to be immunogenic. In these animals, a brief period of expression was followed by abrupt decline to baseline as already noted. Plasma samples taken at 4 to 6 weeks gave a strong signal for anti-BChE antibody, averaging 300 times the near-zero baseline values in untreated control mice (Table 2). The same assay failed to detect a statistically significant signal from mice transduced with the conspecific mutant, mCocH, either during the initial peak of activity (at 3 weeks), or subsequently (3 and 8 months). Although this outcome did not definitively establish a complete absence of immune responses, it indicated that the mutations in mouse BChE were at most weakly immunogenic and relatively inaccessible to the host immune system. This finding has favorable implications for therapeutic applications involving other active-site BChE mutants, as will be discussed later.

**Table 2:** Anti-BChE antibodies detected by immunoprecipitation.

	Untreated controls (*n = *10)	AAV-CMV-hCocH (*n = *9)	AAV-CMV-mCocH (*n = *16)
**Age**	–	1–2 months	1–8 months
**CocH binding** (arbitrary units)	20±13	5,950±1,280***	132±87

Treated mice received AAV viral vector encoding mouse BChE mutated for enhanced cocaine hydrolysis (mCocH), or human BChE with comparable mutations (hCocH). Data represent cocaine hydrolase activity adsorbed by anti-mouse IgG and IgM antibodies coupled to a solid-phase matrix (see Methods). Bound activity is expressed in arbitrary units directly proportional to the amount of cocaine-hydrolyzing enzyme. All mice transduced with the human protein, hCocH, gave a strong signal (***, p<0.001). The 16 mice transduced with the murine equivalent, mCocH, gave no significant signal as a group, though one sample was borderline.

### Dose-response Relations for mCocH Transduction with AAV Vector

**Figure 4:**
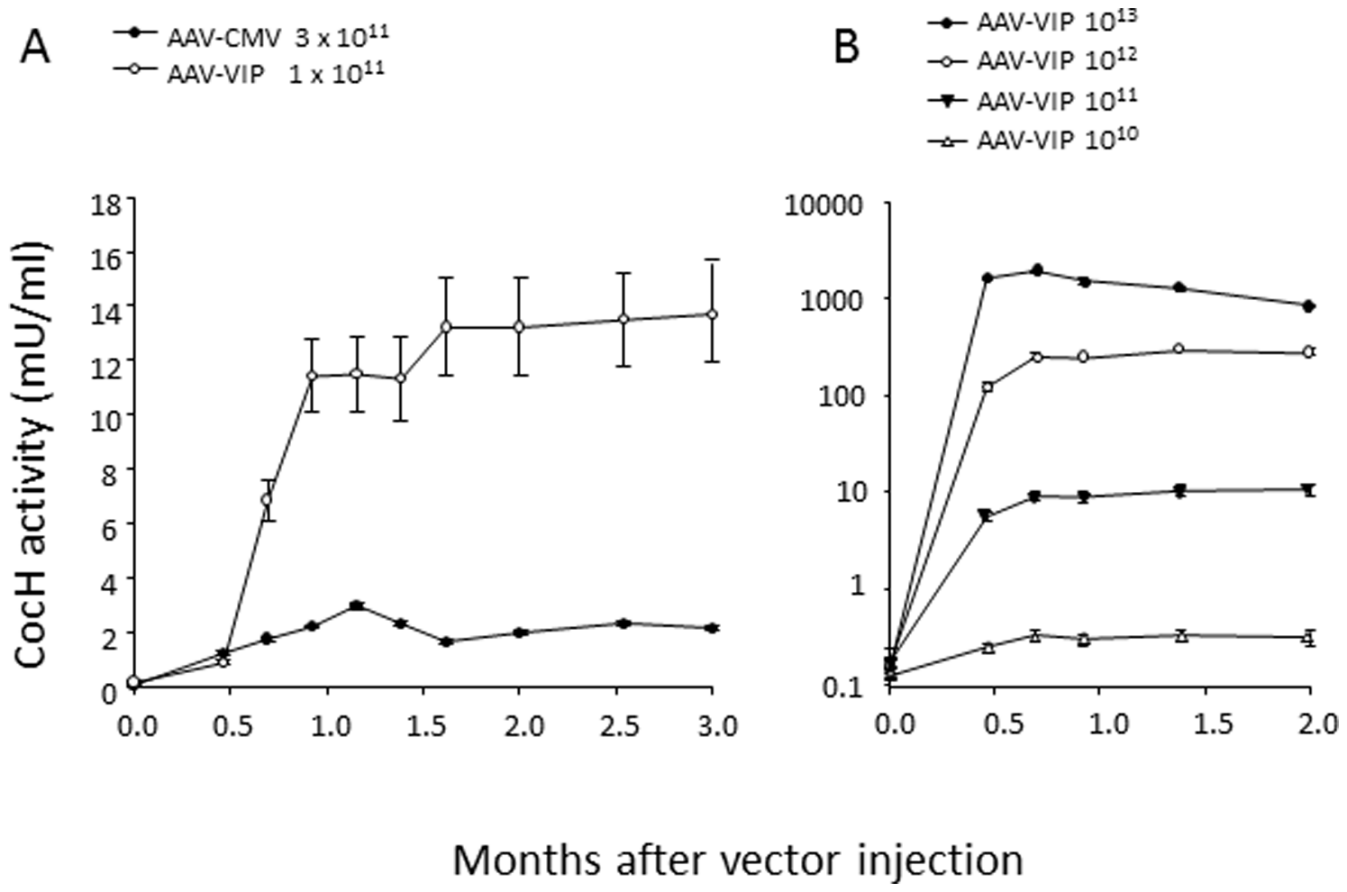
Effect of vector promoter and vector dose on mCocH expression. A) Mice were given injections of AAV-CMV-mCocH vector (3×10^11^ viral particles) or AAV-VIP mCocH vector (10^11^ viral particles). Plasma samples were collected at the indicated time points and assayed for cocaine hydrolysis activity. Means and standard errors of the means are shown (n = 12). B) Mice received AAV-VIP-mCocH vector in the indicated doses, and cocaine hydrolase activity was measured repeatedly from 2 weeks to 2 months. Data, in mU, are reported on a logarithmic scale. Means and standard errors are shown (n = 10 per group).

It was of interest to determine whether enzyme expression with AAV vector could be enhanced with more effective regulatory elements. For that purpose we compared mCocH transduction by AAV-CMV with a recently reported “high efficiency” AAV-VIP vector [13] of the same serotype (see Methods). The results indicated that AAV-VIP at a dose of 10^11^ particles was almost 10-fold more effective than AAV-CMV at equivalent or higher doses (Fig. 4A). Therefore most subsequent experiments with AAV vector utilized this new construct.

**Figure 5:**
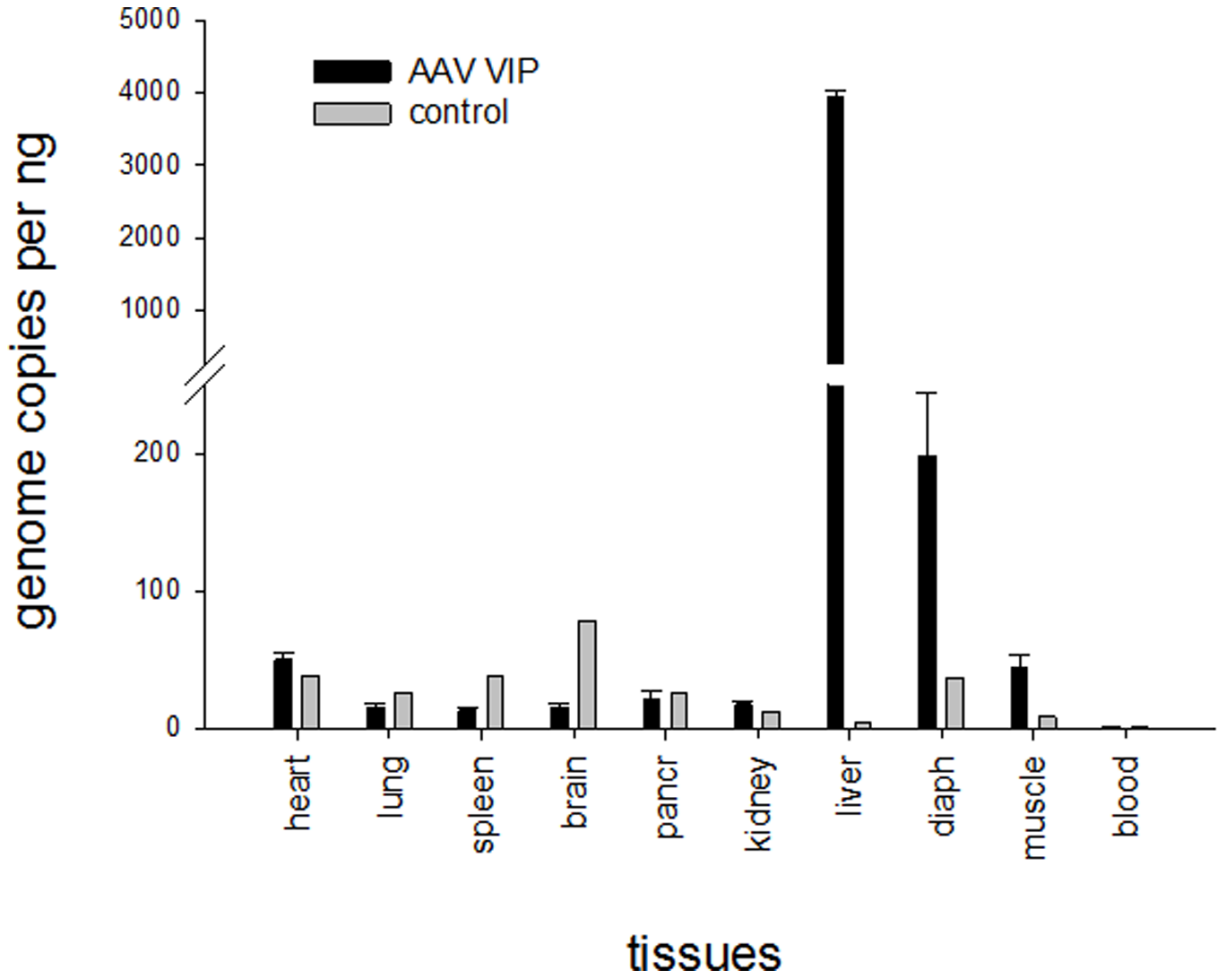
Quantitative PCR evidence for liver as primary site of vector. Mice were euthanized 2 months after i.v. injection of AAV-VIP vector with mutated BChE at a dose of 10^12^ viral particles per animal. Vector DNA in the indicated tissues was quantitated by qPCR and expressed as viral genome copies per ng of host DNA.

When multiple doses of the AAV-VIP mCocH vector were compared, the plasma level of mouse CocH activity showed a strong dose-dependency that was stable over a two-month period of examination (Fig. 4B). At the top dose (10^13^ viral particles) activity was more than 10,000-fold above the level in untreated mice. After this study, mice from one dose group (10^12^ particles) were euthanized, and tissue samples were examined by QPCR to determine viral copy numbers in relation to measured enzyme activities (Fig. 5). Liver was by far the major locus of viral particles (4100±70 copies per ng of genomic DNA) and liver homogenates displayed by far the highest level of CocH activity (80 mU/mg). The next most abundant location was diaphragm muscle (200±38 copies/ng). No significant level of vector was detected in heart, lung, spleen, brain, pancreas, kidney, or whole blood, and the minor amounts of CocH activity in homogenates of these tissues were attributed to blood residues despite aortic perfusion.

### Very High Expression After High-dose hdAD Vector

**Figure 6:**
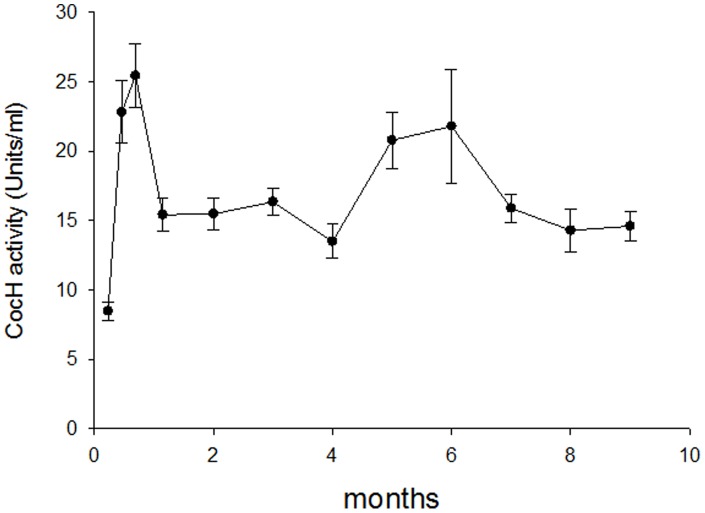
Extreme mCocH expression with high-dose hdAD vector. Mice (n = 10) were given vector injections of 1.7×10^12^ viral particles. Circulating levels of cocaine hydrolase activity are shown (note ordinate scale in units (U) rather than milliunits).

To explore the range of cocaine hydrolase transduction with a different vector platform, ten mice were injected with hdAD-vector encoding the same mouse CocH sequence. Because low doses had proved less than optimal, mice were given 1.7×10^12^ viral particles i.v. Surprisingly, plasma cocaine hydrolase activity rose to a peak value of 25 U/ml (Fig. 6), ∼ 10,000-fold above the 1 to 3 mU/ml obtained in the early AAV experiments and 280,000-fold above the baseline value in untreated control mice. Total activity against butyrylthiocholine rose less, approximately 1000-fold, corresponding to a 1500-fold rise in enzyme protein after allowing for its reduced efficiency with that substrate. From multi-species studies of BChE activity and kinetics [14], [15], [16] we estimated the level of native BChE in normal mouse plasma at 5 to 6 µg/ml, and we therefore calculated a rise to 8 mg/ml in the transduced subjects. The high-level expression of BChE activity was well maintained, remaining near 15 U/ml for the whole observation period (9 months to date). In contrast, measurements of plasma acetylcholinesterase (AChE) showed no disturbance from the control mouse level of 0.5 U/ml.

### Absent Locomotor Response to Cocaine in Hydrolase-transduced Mice

**Figure 7:**
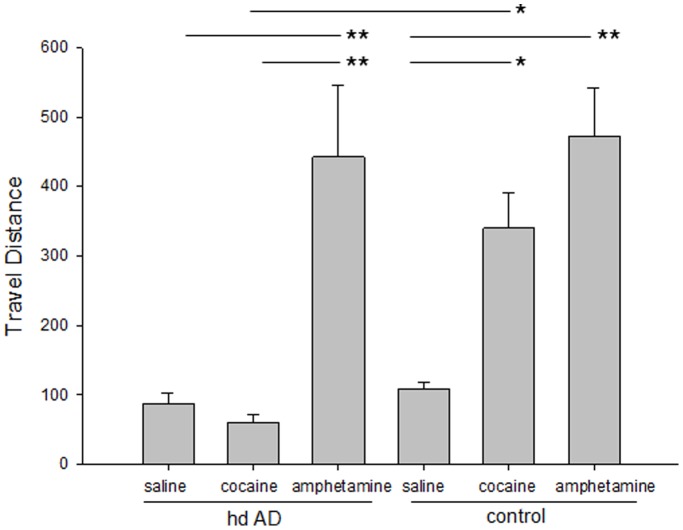
Spontaneous and stimulant-induced locomotor activity in control mice and mice transduced with mCocH by high-dose hdAD vector. Subjects (n = 8 to 12) were tested in locomotor activity chambers 6 weeks after receiving hdAD-mCocH vector. Controls were previously untreated animals. Shown are means and SEM of locomotor activity (cm traveled per 2-min bin) from hour-long sessions on consecutive days beginning immediately after injection of saline (days 1–3), cocaine 40 mg/kg (day 4), and D-amphetamine 5 mg/kg (day 7). One-way analysis of variance indicated a highly significant difference between treatment groups (F = 10.6, p<0.001). Statistical significance of the most relevant significant pairwise multiple comparisons is indicated by asterisks (* t >3.1, p<0.01; ** t >3.8, p<0.001). The complete analysis is available in Supplementary Materials (Table S2).

Our previous experience showed that direct injection of cocaine hydrolase based on human BChE (hCocH) reduced or completely suppressed a wide variety of responses to cocaine [5], [12], [17], [18], [19]. To confirm that over-expression of mCocH acts in similar fashion, mice from the high-dose hdAD group were tested in hour-long sessions in an automated locomotor activity chamber. These animals exhibited no excess ambulation or other behavioral signs when challenged with a strongly stimulating dose, of cocaine, 40 mg/kg, i.p. (Fig. 7). This suppressant effect was specific to cocaine, as the animals responded robustly to the related stimulant, D-amphetamine, not a substrate for mCocH. In a follow-up experiment to probe the limits of protection, the same mice were given cocaine at much higher dosage (120 mg/kg, i.p.). No quantitative locomotor studies were performed in this case, but close observation failed to identify any outward reaction from these mice, such as tremors, gait abnormalities or other signs. In comparison, unprotected mice were prostrated by the overdose and several died.

### Toxicological Studies

Liver is a predominant target both for AAV-VIP and hdAD-ApoE vectors, and most transduction of mutant BChE takes place in that tissue as demonstrated previously [12] and confirmed here. Therefore, in mice given high doses of hdAD or AAV vectors transducing high mCocH expression, we investigated plasma alanine aminotransferase (ALT), a sentinel indicator of liver damage. ALT activities in both vector groups were low normal at 3 weeks (data not shown) and at 3 months (Table 3). Furthermore, 24 hr after these mice received the 120 mg/kg cocaine overdose, their plasma ALT remained at the level of saline treated controls. In comparison, non-transduced animals challenged in the same manner showed a roughly 400-fold increase in ALT activity indicative of severe pathology and hepatic necrosis. Thus by this measure the high dose vector treatments were not only non-toxic themselves, they were able to protect mice against a near-lethal hepatotoxic insult from cocaine.

**Table 3:** Biomarker tests of liver, muscle and heart toxicity in vector-treated animals.

	Saline			Cocaine		
Pretreatment	Saline (*n = *8)	hdAD-mCocH (*n = *8)	AAV-mCocH (*n = *10)	Saline (*n = *8)	hdAD-mCocH (*n = *8)	AAV-mCocH (*n = *10)
**ALT activity**	16.4±8.9	22.1±7.4	21.0±7.9	7900±1560**	20.5±2.4	11.6±0.7
**SkM-Tn-I**	4.6±1.8	3.3±1.3	3.1±0.63	307±180 *	5.3±2.5	1.2±0.2

Mice were pretreated i.v. with one of two viral vectors as indicated: a) hdAD encoding mCocH (1.7×10^12^ viral particles), or b) AAV-VIP encoding the human CocH (10^13^ viral particles). At the same time, control mice received saline injection or no treatment. Three to four months after pretreatment, a saline injection was given and plasma samples were assayed 24 hr later for these biomarkers: ALT (alanine aminotransferase activity, U/L), skeletal muscle troponin-I (SkM-Tn-I, ng/ml), and cardiac troponin-I (C-Tn-I, ng/ml). A week later the same mice were challenged with a large dose of cocaine (120 mg/kg i.p.), and the biomarkers were re-determined after 24 hr. Note the 500-fold rise of plasma ALT in unprotected subjects, ** (p<0.001), and the 66-fold rise of SkM-Tn-I, * (p<0.01 by Mann-Whitney test).

Two months later similar experiments were performed on the same mice in order to evaluate vector toxicity in cardiac and skeletal muscle. For this purpose, plasma levels of troponin-I from skeletal and cardiac muscle were selectively determined by ELISA before and 24 hr after cocaine (Table 3). Before drug challenge both measures were low in the vector-treated groups (mean values non-significantly *below* control). Thus, as with liver, there was no evidence of vector-related toxicity in either of these contractile tissues. After cocaine challenge in saline pretreated mice, skeletal muscle troponin-I rose dramatically, by almost the same multiple as ALT activity (Table 3). The data did not follow a normal distribution but the increase was highly significant (p<0.01) by Mann-Whitney test and by standard t-test after log transformation. This sign of cocaine toxicity was abolished in mice pretreated with either of the CocH-transducing viral vectors. Cardiac-specific troponin I was not detectable in plasma samples from any of the tested groups, including those receiving cocaine with or without vector treatment.

## Discussion

These experiments addressed the potential immunogenicity of the mutations in murine BChE conferring high catalytic efficiency with cocaine. The results indicate that the suite of five mutations under study, optimal or near optimal for cocaine hydrolysis [2], [3], does not constitute an "immunological signature" that provokes a strong antibody response. All but one of 16 mice transduced with viral vector for the pentavalent-mutant transgene, mCocH, developed no antibody detectable with a sensitive pull-down assay (the exception gave a weak reaction). In contrast, the assay readily measured anti-BChE antibodies in virtually all mice transduced with the equivalent human protein. This outcome was anticipated because most of the altered residues in mCocH sit deep in the enzyme's catalytic gorge and may well be shielded from immune surveillance. Nonetheless, these supportive animal data should help establish the clinical safety of a proposed gene transfer therapy with modified human BChE.

The modified mouse enzyme's weak antigenicity probably explains the contrast between the indefinite duration of mCocH transduction versus four weeks for its human equivalent, which provoked an antibody response under present conditions in mice, though not previously in rats [5]. It is also worth noting that this long term expression was matched by good *in vivo* stability of mCocH, judging by the rate of recovery of activity after irreversible inhibition. This outcome is somewhat surprising since the mutant enzyme was expressed primarily in the form of dimers rather than tetramers, the more stable and typically dominant form. One likely reason for the relatively low degree of oligomerization is a limited availability of the proline rich peptides (PPrPs) that are required for cholinesterases to form tetramers [20]. These peptides are more abundant in humans [21]. Therefore, CocH transduction in a clinical setting may lead to more stable tetramers that accumulate to higher levels at any given rate of biosynthesis.

A dramatic finding was the high final level of circulating cocaine hydrolase after a large dose of hdAD vector, associated with a nearly 300,000-fold increase in cocaine hydrolyzing activity and an estimated 1500-fold increase in BChE protein. This increase moved BChE in these animals from "minor" to "major" serum protein, nearly as abundant as albumin. We are unaware of a comparable result in previous gene transfer studies. However, the noticeable difference in enzyme levels with the two vectors probably reflects many factors. Therefore, these data are not a strong basis for determining which vector is best for clinical use. It is critically important to make that determination before entertaining a clinical trial. At present there is a general sense that AAV vectors are safer than hdAD, and a growing number of gene therapy trials rely on this platform [22], [23]. Two crucial questions to resolve in choosing AAV or hdAD for future CocH gene transfer in humans are: 1) Can we shrink the difference in enzyme transduction by these agents at equivalent viral loads? 2) Which will transduce more protein at an acceptable level of toxicity? The data reported here with hdAD and AAV-VIP represent a step towards resolving question one.

Given the impressive expression of mCocH, which also hydrolyzes acetylcholine, the lack of physiological abnormality in mice is remarkable. Our finding of normal spontaneous locomotor activity suggests that very high plasma BChE activity does not impair neuromuscular function. This agrees with prior research on BChE's physiological impact. Thus, studies evaluating human BChE as prophylaxis against chemical warfare agents found no autonomic or motor impairment in rats, guinea pigs or primates, even with gram doses raising plasma enzyme levels 50–100-fold [7], [8], [9], [24], [25], [26]. This result could have been predicted. First, the molar concentrations of acetylcholinesterase (AChE) and BChE in blood (plasma plus red cells) are roughly similar [14] and, as AChE is more catalytically efficient [27], a 10-fold increase in circulating BChE only triples acetylcholine hydrolysis. Second, cholinergic synapses in the brain are insulated from plasma enzymes by the blood-brain barrier. Third, peripheral cholinergic synapses are densely packed with AChE. Anglister's classical morphometric measures of cholinesterase density using ^125^I-fasciculin labeling indicated 5×10^19^ catalytic AChE subunits per cc (∼ 0.1 mM) in mouse neuromuscular junction [28]. In comparison mouse plasma BChE levels are below 0.1 μM [14]. Since BChE hydrolyzes acetylcholine half as efficiently as AChE, thousands-fold more would be needed to match the synaptic capacity for ACh-hydrolysis. Hence, even high levels of plasma BChE are unlikely to affect motor transmission. Remaining areas for potential concern are nicotinic synapses in peripheral autonomic ganglia and muscarinic synapses with parasympathetic innervation. Investigations of cardiovascular function in vector-treated mice are underway to address such issues.

ALT and troponin-I screening in mice with abundant mCocH levels revealed no adverse effects of gene transduction *per se* in liver, skeletal muscle or heart and indicated protection of liver and skeletal muscle from large cocaine doses. That outcome matches our previous findings with direct delivery or gene transfer of *human* CocH [29]. The similar levels of protection were surprising since hCocH hydrolyzes cocaine more efficiently than mouse CocH [2], [3]. However, total plasma enzyme concentration was substantially greater in the current experiments. Current data do not allow us to conclude that mCocH gene transfer will also protect against cocaine-induced cardiotoxicity, because the cocaine challenge did not affect cardiac troponin-I in unprotected mice. Ongoing cocaine experiments in vector-naïve mice, however, confirm that *repeated* dosing will cause measureable release of troponin-I from heart. These unpublished results serve as a positive control for further experiments to determine whether vector treatment will prevent cardio-toxicity.

If gene transduction of CocH or a similar enzyme reaches clinical trial or enters clinical practice, it must use lower levels of viral vector. The main reason will be to avoid acute toxicity from agents that, even though nonpathogenic and non-replicating, contain viral antigens that provoke host immune reactions [30], [31]. Lower levels of vector imply lower enzyme expression. Therefore, the present results indicate that expected levels of BChE expression will pose no real added risk. The important question is whether those levels will reduce responses to cocaine and lower its reward value sufficiently to be therapeutic. If not, there is still reason for optimism that cocaine hydrolase gene therapy combined with anti-cocaine vaccine will be helpful to recovering cocaine addicts.

There is a large literature on vaccines against drugs of abuse. Such vaccines can generate substantial titers of high-affinity antibodies against psychoactive small molecule haptens conjugated to appropriate carrier proteins such as keyhole limpet hemocyanin (KLH), cholera toxin (CTB) [32], and tetanus toxoid (TTX) [33]. Moderately encouraging results have been obtained with vaccines to cocaine, as well as heroin, nicotine, and methamphetamine [34], [35], [36], [37]. Thus, several studies have found reduced drug levels in plasma or brains of rodents with high titers of specific drug antibody [38], [39], [40], [41]. A clinical trial of anti-cocaine vaccine indicated partial therapeutic effects in the form of increased frequency of drug free urines [42]. Such effects were related to the antibody titers, which varied substantially among individuals, precluding a statistically significant effect overall. Better results should follow further improvements in vaccine technology.

Recent studies, in collaboration with Kosten and Orson at Baylor, supported the concept of synergy between these two treatment modalities. In particular, the combination of anti-cocaine antibodies elicited by a nor-cocaine conjugate vaccine [43] and cocaine hydrolase delivered either by direct i.v. enzyme injection or by gene transfer proved particularly effective in preventing muscle weakness and liver damage [29]. The same combination also was super-additive in reducing stimulatory locomotor responses and the behavioral sensitization that typically follows repeated cocaine exposures [19]. In a recent review article [1] we discussed the advantages and limitations of such treatments in greater depth, including possible susceptibility to higher drug doses and reduction of overdose risk. In our view, the accumulated data strongly support continued efforts toward developing enzyme-based gene transfer therapies along with more effective vaccines for cocaine abuse.

## Materials and Methods

### Animal Subjects and Ethics Statement

Adult male mice (Balb/c), ∼ 25 g in weight, were obtained from Harlan Laboratories, Madison Wisconsin, under protocol A26810 (approved by the Mayo Clinic Institutional Animal Care and Use Committee). All experiments were conducted in accordance with the Guide for Care and Use of Laboratory animals [44] in a facility accredited by the American Association for the Accreditation of Laboratory Animal Care. Drugs were injected i.p. in isotonic saline solution. Viral vectors were delivered by rapid injection through the tail vein in an initial volume of 200 µl followed by 200 µl of 0.9% sterile NaCl solution.

### Chemicals, Drugs, and Reagents

Cocaine HCl was obtained from the National Institute of Drug Abuse (Research Triangle Institute, Research Triangle Park, NC USA). This drug was freshly dissolved in 0.9% NaCl for each mouse experiment at a concentration that allowed delivery of 200 µl per 30 g (typical subject weight). D-amphetamine sulfate, butyrylthiocholine iodide, tetraisopropylphosphoramide (iso-OMPA), diisopropylfluorophosphate (DFP), and other reagents including Pansorbin were purchased from Sigma-Aldrich (St Louis MO).

### Blood Collection, Enzyme Assay and Cocaine Determinations

Blood samples (<0.1 ml) were taken from mice by cheek puncture using a 21-gauge mouse-bleeding lancet. A sterile gauze pad was then applied with slight pressure for about a minute to stop bleeding. Blood samples were centrifuged for 15 min in serum separator tubes (Becton Dickenson, Franklin Lakes, NJ, USA) at 8000 g and the sera were stored at −20° C before analysis of antibody and CocH enzyme levels. Cocaine hydrolase activity in duplicate 50-µl aliquots of serum was assayed by incubating 30 min with ^3^H-cocaine (50 nCi, 18 µM; Perkin Elmer, Boston MA) and measuring liberated ^3^H-benzoic acid after acidification and partition into toluene-based fluor for scintillation counting as described previously [45].

### Viral Gene Transfer

Mutations of enzyme cDNA were initiated with a method based on adeno-associated virus as previously described [46], [47], [48]. First, wild type mouse BChE cDNA (provided by S. Camp and P. Taylor, UCSD) was cloned into a pAAV-CMV shuttle plasmid for serotype 8 adeno-associated virus (AAV) gene transfer vector. A Kozak consensus sequence (GCCACC) was introduced above the translational start site. With this construct as template, site-directed mutagenesis using primers with specific base-pair alterations generated the mouse BChE mutant here termed mCocH, A199S/S227A/S287G/A328W/Y332G (for primer sequences see supplementary material, Table S1). The mCocH DNA was also incorporated into a newly reported “AAV-VIP" vector” [13] with enhanced transduction efficiency from a CMV enhancer, a chicken beta-actin promoter and a ubiquitin promoter. To construct the plasmids for mCocH, recognition sites for “Not I” and “BamH I” were introduced directly before the Kozak consensus sequence and after the stop codon, respectively. Subsequently, mCocH was ligated into a "pAAV-VIP" plasmid between Not I and BamH I.

To produce and purify AAV8 viral particles, the plasmids pAAV-CMV-BChE (wt or mCocH) or pAAV-VIP-mCocH were co-transfected into HEK293T cells with helper vectors, pHelper and pAAV2/8, using FuGene HD Transfection Reagent (Roche). Three days later, AAV8 virus was purified from the cell lysates by ultracentrifugation against Optiprep Density Gradient Medium-Iodixanol (Sigma-Aldrich, St Louis MO). The concentration of viral particles was subsequently determined by real-time quantitative PCR (QPCR), which was also used to establish the tissue distribution of delivered vector.

The mCocH cDNA was also incorporated into a serotype-5 helper dependent adenoviral vector (hdAD) under regulation by a human ApoE hepatic control region [49], with a bovine growth hormone polyadenylation sequence cloned into a derivative of the p28lacZ hdAD-backbone plasmid. Vector was propagated using the AdNG163 helper virus, as described [50], and particle titers were determined by optical density at 260 nm. Helper virus contamination, determined by plaque assay on HEK-293 cells, was ∼ 0.2% for both loaded and empty vectors.

### Determining Viral Copy Numbers in Mouse Tissues

Total DNA was isolated from heart, lung, spleen, pancreas, brain, kidney, diaphragm, hind limb muscle, liver and whole blood using the QIAamp DNA Mini Kit (Qiagen, Hilden, Germany) and quantitated by absorbance at 260 nm. Real time polymerase chain reaction assays (QPCR) were performed in a 20-μl volume comprising 1X iQ SYBR Green Supermix (BIO-RAD), 10 ng of tissue DNA and 0.1 μM of each AAV-VIP-specific primer (forward: 5′-AACGCCAATAGGGACTTTCC-3′; reverse: 5′-GGGCGTACTTGGCATATGAT-3′). The PCR profile was 95°C for 3 min followed by 39 cycles of 95°C for 10 sec and 55°C for 30 sec. A standard curve was generated using serial dilutions of pAAV-VIP-mCocH plasmid DNA to determine genome copies of AAV8-VIP-mCocH in tissues and the results were analyzed with SDS2.3 software (Applied Biosystems, Foster City, CA).

### In vitro Enzyme Expression and Enzyme Purification and Substrate Kinetics

Three days after transfecting HEK293T cells with AAV-plasmid encoding mCocH or wild type mouse BChE, enzyme was isolated from the culture supernatants by procainamide-Sepharose gel chromatography (gift from Dr. O. Lockridge, Univ. Nebraska), followed by ion-exchange chromatography. Purification led to a single major band on SDS polyacrylamide gel electrophoresis (Fig. 1). Active sites were titrated with DFP to determine final molar enzyme concentration as described previously [51]. Cocaine substrate kinetics were tested across a wide range of concentrations using the radiometric assay described above. Comparable experiments with acetyl- and butyrylthiocholine used the classic Ellman spectrophotometric assay [52]. To estimate V_max_, K_m_, K_ss_, and “b”, kinetic data were fitted to a modified Michaelis-Menten equation with Sigma Plot (Version 11.0, Systat Software) as described [15], [16].

### Detection of CocH Antibodies

Immunoadsorption with Protein-A-bearing *Staphylococcus aureus* cells (“Pansorbin”, EMD Biosciences Inc., La Jolla CA) was used to screen for IgG or IgM antibodies to mCocH. Linker antibodies against mouse IgG and IgM (Thermo Scientific, Rockford IL) at a concentration of 0.24 mg/ml were adsorbed to Pansorbin aliquots for 1 hr at 37°C. After buffer rinsing the pellets were re-suspended and exposed 1 h at 37°C to 100 µl plasma samples plus 150 µl of 50 mM sodium phosphate, pH 7.4, followed by 3 cycles of buffer washes and centrifugation at 2000 g for 10 min. These preparations along with any adsorbed mouse antibody were then tested for ability to bind or inactivate cocaine-hydrolyzing protein during a 1 h exposure to a standard sample of transduced plasma. Binding was evaluated by measuring the loss of cocaine hydrolase in supernatants and appearance of that activity in pellets re-suspended after a final centrifugation at 2000 g. Bound hydrolase activity versus ^3^H-cocaine as substrate was arbitrarily expressed as counts per min (cpm) per 100 µl sample in a 30 min assay.

### Biomarker Assays for Vector-driven Toxicity in Liver, Heart, and Skeletal Muscle

Liver toxicity was evaluated from plasma levels of alanine transaminase (ALT) activity, measured spectrophotometrically by the modified Wrobleski assay [53] with kit TR71121 from Thermo scientific, Middletown, VA. In brief, mouse sera with or without dilution were mixed with assay reagents (L-alanine and 2-oxolglutarate) in the presence of lactic acid dehydrogenase and NADH. Sample absorbance was then read at 340nm for 10 min in a Molecular Dynamics Plate Reader. Activity above blank (saline in place of serum) was expressed in U/L. Toxicity in heart and skeletal muscle was evaluated by enzyme-linked immunoassays specific for the forms of troponin-I in each tissue, using spectrophotometric kits with horseradish peroxidase detection (KT-470 and KT 472, Kamiya Biomedical Company, Seattle, WA). Data were obtained in ng/ml.

### Statistical Methods

Normally distributed data were analyzed by standard t-test or one way analysis of variance with post-hoc testing by Fisher's PLSD. Data that failed preliminary tests of normality were analyzed by the non-parametric Mann-Whitney test and by t-test after conversion to a log scale. With the behavioral data, a one way analysis of variance was conducted and, in light of the high significance of treatment effects, all possible pairwise comparisons were examined by the Holm-Sidak method, using an overall significance level of 0.05.

## Supporting Information

Table S1 **Oligomeric primers for mutagenesis of mouse butyrylcholinesterase.** The indicated primer pairs were used to make specific mutations in mouse BChE to generate a murine enzyme containing the identical pentameric suite of active site amino acids previously found to confer optimal catalytic efficiency in cocaine hydrolysis by human BChE.
(DOCX)

Table S2 **Detailed statistical analysis of locomotor behavior.** All possible pairwise comparisons of locomotor behavior in different treatment groups were analyzed by analysis of variance. Key points to note are: 1) highly significant effects of treatment with hdAD vector on responses to cocaine but not on responses to amphetamine; 2) lack of significant difference between hdAD-treated mice given cocaine and control mice given saline; 3) lack of difference between control mice given saline and hdAD treated mice given saline.
(DOCX)
