# Supplementary material for: Gene Transfer of Mutant Mouse Cholinesterase Provides High Lifetime Expression and Reduced Cocaine Responses with No Evident Toxicity
Source: PLoS One. 2013 Jun 28;8(6):e67446. doi: 10.1371/journal.pone.0067446 (PMC3696080; doi:10.1371/journal.pone.0067446)
Supplement: Table S1 — Oligomeric primers for mutagenesis of mouse butyrylcholinesterase. The indicated primer pairs were used to make specific mutations in mouse BChE to generate a murine enzyme containing the identical pentameric suite of active site amino acids previously found to confer optimal catalytic efficiency in cocaine hydrolysis by human BChE. (DOCX) [file pone.0067446.s001.docx]

**A328WY332G:**

**5’ GGGAGTTAACAAAGATGAAGGGACATGGTTCCTAGTGGGCGGTGCTCCGGGTTTCAGC3’**

**5’-GCTGAAACCCGGAGCACCGCCCACTAGGAACCATGTCCCTTCATCTTTGTTAACTCCC3’**

**A199S:**

**5’ ACGATTTTTGGAGAAAGTTCAGGGGCAGCTTCAGTTAGC3’**

**5’ GCTAACTGAAGCTGCCCCTGAACTTTCTCCAAAAATCGT3’**

**S287G:**

**5’CCCCTCTGATTCCATCTTAGGCATAAATTTTGGTCCAACAGTGG3’**

**5’CCACTGTTGGACCAAAATTTATGCCTAAGATGGAATCAGAGGGG3’**

**S227A:**

**5’GAGCCATTCTTGAAAGTGGCTCCGCTAATGCCCCCTGGGCAG3’**

**5’CTGCCCAGGGGGCATTAGCGGAGCCACTTTCAAGAATGGCTC3’**
