# Supplementary material for: Gene Transfer of Mutant Mouse Cholinesterase Provides High Lifetime Expression and Reduced Cocaine Responses with No Evident Toxicity
Source: PLoS One. 2013 Jun 28;8(6):e67446. doi: 10.1371/journal.pone.0067446 (PMC3696080; doi:10.1371/journal.pone.0067446)
Supplement: Table S2 — Detailed statistical analysis of locomotor behavior. All possible pairwise comparisons of locomotor behavior in different treatment groups were analyzed by analysis of variance. Key points to note are: 1) highly significant effects of treatment with hdAD vector on responses to cocaine but not on responses to amphetamine; 2) lack of significant difference between hdAD-treated mice given cocaine and control mice given saline; 3) lack of difference between control mice given saline and hdAD treated mice given saline. (DOCX) [file pone.0067446.s002.docx]

| Normality Test (Shapiro-Wilk) | Failed | (P < 0.050) | | |  |  |
| --- | --- | --- | --- | --- | --- | --- |
|  |  |  | |  |  |  |
| Equal Variance Test: | Failed | (P < 0.050) | | |  |  |
|  |  |  | |  |  |  |
| Group Name | N | Missing | | Mean | Std Dev | SEM |
| hd ad saline | 10 | 0 | | 87.8 | 46.6 | 14.72 |
| control saline | 19 | 0 | | 107.579 | 43.4 | 9.96 |
| hd ad cocaine | 10 | 0 | | 61.3 | 33.9 | 10.72 |
| control cocaine | 19 | 3 | | 340 | 203.8 | 50.94 |
| hd ad amphetamine | 10 | 0 | | 442.7 | 322.0 | 101.82 |
| control amphetamine | 22 | 0 | | 471.818 | 328.8 | 70.11 |
|  |  |  | |  |  |  |
| Source of Variation | DF | SS | | MS | F | P |
| Between Groups | 5 | 2540502 | | 508100 | 10.58 | <0.001 |
| Residual | 81 | 3890514 | | 48031 |  |  |
| Total | 86 | 6431016 | |  |  |  |
|  |  |  | |  |  |  |
| Differences in mean values among treatment groups are greater than expected by chance (P = <0.001). | | | | | | |
|  |  |  | |  |  |  |
| Power of performed test with alpha = 0.050: 1.000 | |  | |  |  |  |
|  |  |  | |  |  |  |
| Comparisons for factor: | Mean Diff. | t | Unadjusted P | | Critical Level | Significant? |
| control amphetamine vs. con saline | 364.2 | 5.307 | <0.001 | | 0.003 | Yes |
| control amphetamine vs. hd ad cocaine | 410.5 | 4.911 | <0.001 | | 0.004 | Yes |
| control amphetamine vs. hd ad saline | 384.0 | 4.594 | <0.001 | | 0.004 | Yes |
| hd ad amphetamine vs. con saline | 335.1 | 3.914 | <0.001 | | 0.004 | Yes |
| hd ad amphetamine vs. hd ad cocaine | 381.4 | 3.891 | <0.001 | | 0.005 | Yes |
| hd ad amphetamine vs. hd ad saline | 354.9 | 3.621 | <0.001 | | 0.005 | Yes |
| control cocaine vs. hd ad cocaine | 278.7 | 3.155 | 0.002 | | 0.006 | Yes |
| control cocaine vs. con saline | 232.4 | 3.125 | 0.002 | | 0.006 | Yes |
| control cocaine vs. hd ad saline | 252.0 | 2.855 | 0.005 | | 0.007 | Yes |
| control amphetamine vs. con cocaine | 131.8 | 1.831 | 0.071 | | 0.009 | No |
| hd ad amphetamine vs. con cocaine | 102.7 | 1.162 | 0.248 | | 0.01 | No |
| control saline vs. hd ad cocaine | 46.3 | 0.541 | 0.59 | | 0.013 | No |
| control amphetamine vs. hd ad amphet | 29.1 | 0.348 | 0.728 | | 0.017 | No |
| hd ad saline vs. hd ad cocaine | 26.5 | 0.271 | 0.788 | | 0.025 | No |
| control saline vs. hd ad saline | 19.8 | 0.231 | 0.818 | | 0.05 | No |
